# A Potential Neural Substrate for Processing Functional Classes of Complex Acoustic Signals

**DOI:** 10.1371/journal.pone.0002203

**Published:** 2008-05-21

**Authors:** Isabelle George, Hugo Cousillas, Jean-Pierre Richard, Martine Hausberger

**Affiliations:** Université Rennes 1, CNRS, UMR 6552 Ethologie Animale et Humaine, Rennes, France; Centre de Recherches su la Cognition Animale-Centre National de la Recherche Scientifique and Université Paul Sabatier, France

## Abstract

Categorization is essential to all cognitive processes, but identifying the neural substrates underlying categorization processes is a real challenge. Among animals that have been shown to be able of categorization, songbirds are particularly interesting because they provide researchers with clear examples of categories of acoustic signals allowing different levels of recognition, and they possess a system of specialized brain structures found only in birds that learn to sing: the song system. Moreover, an avian brain nucleus that is analogous to the mammalian secondary auditory cortex (the caudo-medial nidopallium, or NCM) has recently emerged as a plausible site for sensory representation of birdsong, and appears as a well positioned brain region for categorization of songs. Hence, we tested responses in this non-primary, associative area to clear and distinct classes of songs with different functions and social values, and for a possible correspondence between these responses and the functional aspects of songs, in a highly social songbird species: the European starling. Our results clearly show differential neuronal responses to the ethologically defined classes of songs, both in the number of neurons responding, and in the response magnitude of these neurons. Most importantly, these differential responses corresponded to the functional classes of songs, with increasing activation from non-specific to species-specific and from species-specific to individual-specific sounds. These data therefore suggest a potential neural substrate for sorting natural communication signals into categories, and for individual vocal recognition of same-species members. Given the many parallels that exist between birdsong and speech, these results may contribute to a better understanding of the neural bases of speech.

## Introduction

One of the most basic questions of cognitive science is how do organisms sort the objects of the world into categories? Categorization is essential to all cognitive processes. Without categorization, each object would be perceived as unique and no generalization rules could be used to take rapid and appropriate decisions [1]. However, because no single perceptual feature is likely to be a necessary and sufficient condition for category membership [2], identifying the neural substrates underlying categorization processes is a real challenge.

One example of categorical perception is the perception of phonemes in human speech [3]. However, categorical perception is specific neither to humans nor to speech. Indeed, chinchillas have been shown to categorically perceive speech much the same way as humans do [4]. There has also been evidence of categorical perception of species-specific vocalizations in monkeys [e.g. 5] and in avian species [e.g. 6].

Among birds, songbirds are particularly interesting because, without necessarily requiring categorical perception *per se*, birdsongs often provide researchers with clear examples of categories of signals allowing species, population and individual recognition. For example, swamp sparrows sing two note categories with different roles in song construction, and there has been evidence of categorical perception for these notes by this species [7]. Budgerigars, which, like songbirds, are vocal learners, have been shown to group vocal stimuli according to functional and acoustical categories [6].

These birds also have the advantage of possessing a system of specialized brain structures found only in birds that learn to sing: the song system [8]. Given the many parallels that exist between birdsong learning and speech development [9]–[11], this system has become a choice model for studying the neural bases of vocal communication [12]. Nuclei involved in song production have thus been well characterized [e.g. 13] but less is known about areas involved in song perception and discrimination [for a recent review, see 14]. Recently however, the caudo-medial telencephalon has emerged as a plausible site for sensory representation of birdsong.

Caudo-medial telencephalon contains thalamo-recipient Field L2, which is comparable to thalamo-recipient layer IV of the mammalian auditory cortex, and two of its targets, caudo-medial nidopallium (NCM) and caudo-medial mesopallium (CMM), which can be compared to supragranular cortical layers [15]. Based on electrophysiological responses [16]–[19] or on the expression of an immediate early gene (IEG–ZENK) [20]–[22], NCM auditory responses have been shown to be the strongest for conspecific songs, followed by heterospecific songs and non-song acoustic signals, and they are known to show a rapid and long-lasting habituation effect that is song-specific. NCM neurons thus appear to be able to discriminate between different conspecific songs, a property that is required for perceptual song discrimination. Moreover, the ZENK response to a social stimulus has been observed to be proportional to the animal's preference for this stimulus: for example in female European starlings, where a preference for male long bout songs over male short-bout songs has been observed, NCM appears to show higher expression of ZENK in response to long-bout than to short-bout songs, independently of the total amount of song that the females heard [23]. It has therefore been suggested that NCM may serve as a common source for behaviourally relevant distinction among conspecific song features, which would be then extracted by different higher processing areas [see also 24]–[27].

**Figure 1:**
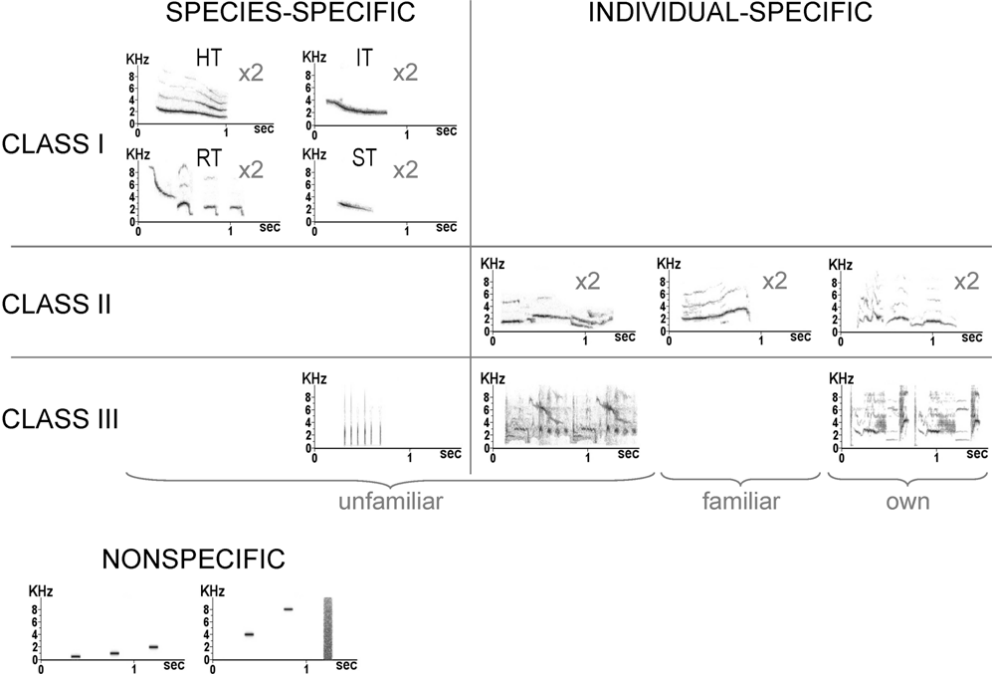
Stimuli used to test the neurons, with some examples of sonograms. Class-I songs are whistles that are produced by all male starlings and that are used in species and population recognition, as confirmed by playback experiments with dialectal variants [29], [30]. Among these songs, 4 themes can be distinguished (from left to right and from top to bottom): harmonic themes (HT), inflection themes (IT), rhythmic themes (RT), and simple themes (ST). Two unfamiliar variants of each theme were used. Class-II songs are individual-specific whistles that are used in long-distance recognition, and that can be shared, in captivity, by a few socially affiliated birds [31]. Two exemplars from the tested bird (own), 2 unfamiliar and 2 familiar exemplars were used. A familiar exemplar was a song produced by a bird that had been caught at the same time as the tested bird, and that had spent 2 years in the same aviary. Class-III songs, also called warbling, are mainly composed of highly individual motifs but also of some motifs common to all starlings [32], [33]. They are used in short distance communication. One species-specific motif (clicks) and two individual-specific motifs (one unfamiliar and one from the tested bird) were used. Finally, 5 pure tones (0.5, 1 , 2, 4 and 8 kHz) and a white noise were used as artificial non-specific stimuli. The stimuli (n = 23) were the same for all the birds except those corresponding to the bird's own songs and to the familiar songs, which changed from one bird to another.

Previous studies thus point to NCM as a well positioned brain region for categorization of songs with different functions and social values. For this reason, we hypothesized that, in our model songbird, which is the starling, NCM neurons may respond differentially to the distinct functional classes of songs that have been described in this highly social species. Indeed, male starlings sing three structurally and functionally distinct classes of songs that are used for species, population and individual recognition (see Fig. 1 for examples) [28]. Class-I songs are short, simple and loud whistles sung by all male starlings that are used in species and population recognition (dialectal variants) [29], [30]. Class-II songs are also short, simple and loud whistles that are used in individual recognition, especially between same-sex social partners [31]. Finally, class-III songs, also called warbling, are long, complex and soft songs that are used in individual recognition at short distance, especially between males and females [32], [33]. These three classes of songs differ not only by their structure [34], [35], but also by their pattern of acquisition during song learning [36]–[38], and by their context of emission [29], [39]–[42]. They thus correspond to clear and distinct classes of sounds with different functions and social values. Hence, we used these songs to test for potential differential responses in the NCM of awake-restrained adult male starlings and for a possible correspondence between these responses and the functional aspects of songs. Our study demonstrates that the activity of NCM neurons can indeed indicate or represent a class of sounds corresponding to a behaviourally-defined recognition process.

## Results

Using a multi-electrode array and a mapping method based on systematic recordings (no search stimulus; see Material and Methods), we recorded the electrophysiological activity of 1972 neuronal sites in the NCM of 6 awake-restrained adult male starlings (mean±SE = 328.7±21.4 sites/bird; 188.2±26.9 sites in the left hemisphere, 140.5±18.3 sites in the right hemisphere; Wilcoxon, p = 0.07), while broadcasting artificial non-specific sounds (pure tones and white noise) and natural species-specific stimuli corresponding to the three classes of songs described in starlings (see Fig. 1). Among these sites, 32.3% were responsive to at least one of the stimuli we used (mean±SE = 31.4±7.4% in the left hemisphere, 35.6±6.5% in the right hemisphere; Wilcoxon, p = 0.25). Only these responsive sites (n = 633; mean±SE = 105.5±22.2 responsive sites/bird) were further analyzed.

**Figure 2:**
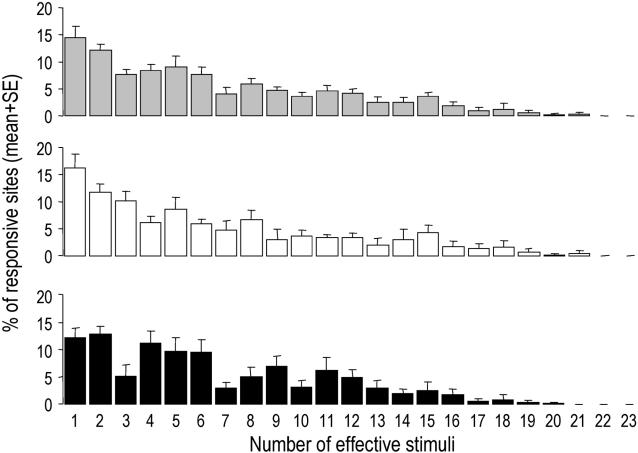
Mean (+SE) percentage of responsive sites that responded to 1, 2, 3… or 23 (that is all) stimuli. Grey bars: pooled data of both hemispheres; white bars: data of the left hemisphere; black bars: data of the right hemisphere.

The relative frequency distribution of sites responding to 1, 2, 3,… or 23 (that is all) stimuli is shown in Figure 2. On average, responsive sites responded to 6.5±0.6 stimuli (mean±SE = 6.5±0.8 stimuli in the left hemisphere, 6.5±0.6 in the right hemisphere; Wilcoxon, p = 0.34), that is about 30% of the stimuli used. About 30% of the responsive sites responded to only 1 or 2 stimuli, and no site responded to all stimuli. Overall, more than 70% of the sites that responded to only one stimulus responded to class-II or –III stimuli, and less than 10% responded only to nonspecific stimuli (note that we could observe no site responding only to the 4- and 8-kHz pure tones and to the white noise).

**Figure 3:**
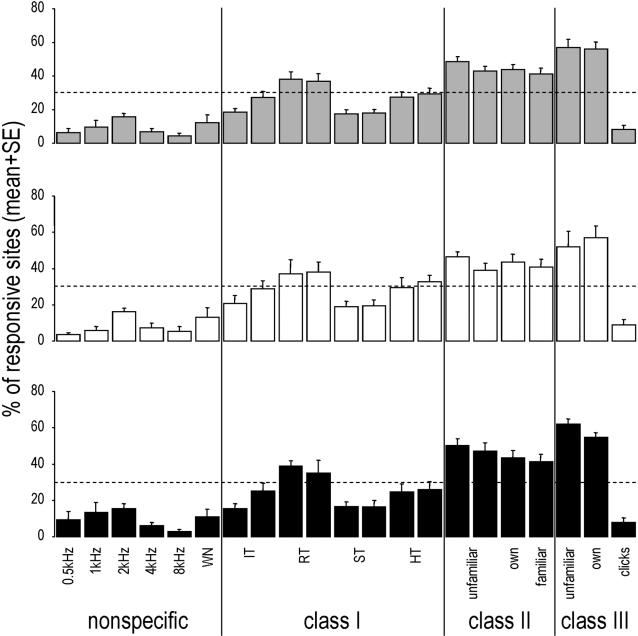
Mean (+SE) percentage of responsive sites that responded to each stimulus. Grey bars: pooled data of both hemispheres; white bars: data of the left hemisphere; black bars: data of the right hemisphere. As the familiar and bird's own class-II songs changed from one bird to another, the data obtained for the two bird's own whistles and for the two familiar whistles were combined. The dashed lines correspond to the uniform expected distributions calculated using the standard method of predicting that the same proportion of sites (weighed by the mean number of stimuli to which they responded) will respond to each stimulus.

When responses to each stimulus were considered, it appeared that, globally, many more sites responded to natural species-specific sounds than to artificial non-specific stimuli (with overall 4.2–15.7% of the responsive sites responding to artificial non-specific stimuli vs. 8.3–56.9% responding to natural species-specific sounds; see Fig. 3). However, the different species-specific song types were not equally effective at driving neuronal responses, and some of them even elicited responses at a level similar to some artificial non-specific sounds, such as some of the class-I songs (the simple themes, with 17.5 and 18.0% of the responsive sites responding; Fig. 3) and the species-specific class-III motif (clicks, with 8.3% of the responsive sites responding; Fig. 3). In fact, most sites responded to songs used in individual recognition, such as the class-II whistles (with 41.0–48.4% of the responsive sites responding; Fig. 3) and the individual-specific class-III motifs (with 56.1 and 56.9% of the responsive sites responding; Fig. 3).

**Figure 4:**
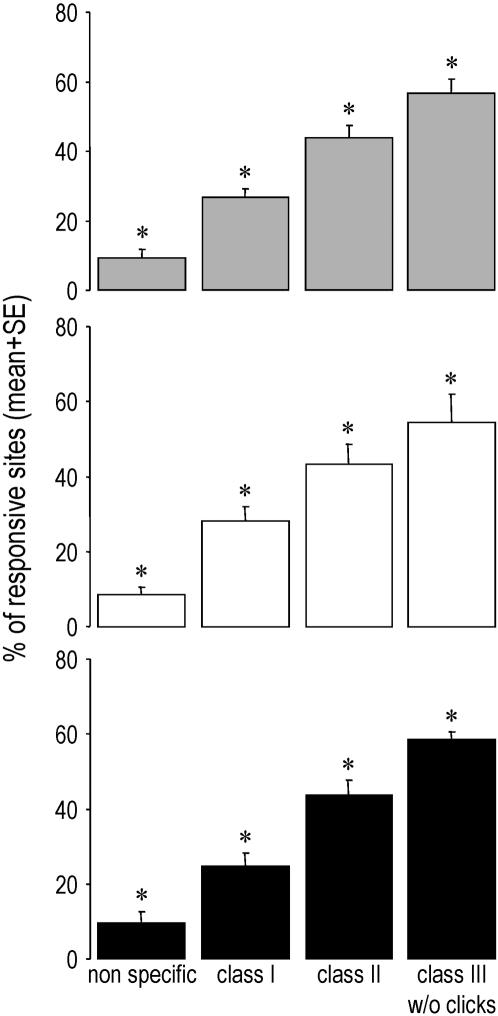
Mean (+SE) percentage of responsive sites that responded to each class of stimulus. Grey bars: pooled data of both hemispheres; white bars: data of the left hemisphere; black bars: data of the right hemisphere. * p<0.001 compared to every other groups (PLSD Fisher tests).

**Figure 5:**
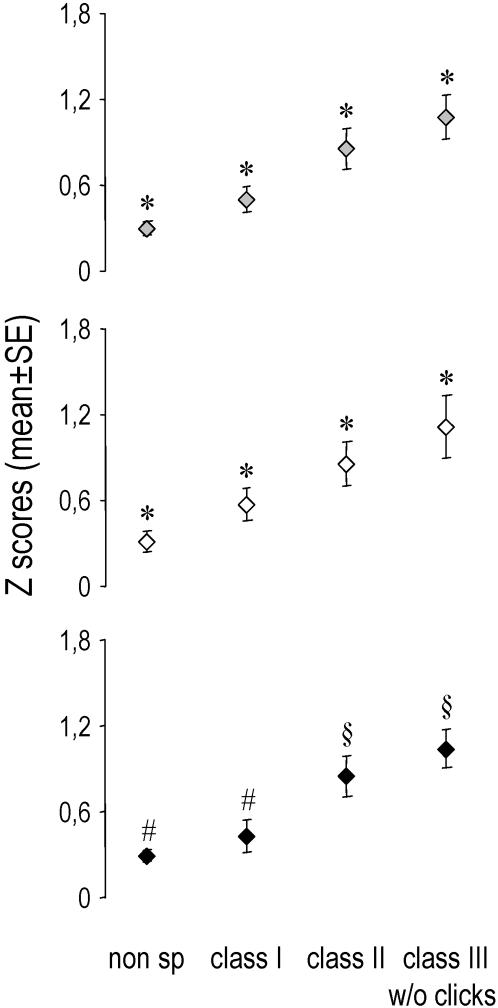
Mean (±SE) Z scores obtained for each class of stimulus. Grey bars: pooled data of both hemispheres; white bars: data of the left hemisphere; black bars: data of the right hemisphere. * p<0.05 compared to every other groups (PLSD Fisher tests). # p<0.01 compared to class II and class III w/o clicks (PLSD Fisher tests). § p<0.01 compared to non specific and class I (PLSD Fisher tests).

Most importantly, when we considered responses to each class of stimuli, it appeared that both the proportion of responsive neuronal sites (Fig. 4) and the magnitude of the neuronal responses (as measured by Z scores; Fig. 5) significantly differed from one class to another: responses were the strongest for the highly individual class-III motifs, followed by the individual-specific class-II songs, the species-specific class-I whistles, and finally the artificial non-specific stimuli (two-way repeated-measures ANOVAs and PLSD Fisher tests, stimulus class effect: p<0.0001 for both the proportion of responding sites and the Z scores, post-hoc comparisons: p<0.05 for all pairwise comparisons in both cases, no hemisphere effect, no interaction). These differences were neither due to a specific bird (since within-bird comparisons showed the same effect or trend in each bird) nor to one particular subset of stimuli (as, with the exception of the species-specific clicks in class III, proportions of sites responding to each stimulus appeared to be relatively homogenous within each class; see Fig. 3). Thus, intra-class variations appeared to be lower than inter-class variations, especially for class-II and individual-specific class-III stimuli which showed coefficients of variation (CVs) that were 3 to more than 7 times lower than the CV observed across all stimuli (mean CVs for class II = 21 and 22%, for class III w/o clicks = 12 and 9% and for all stimuli = 64 and 69%, respectively for the left and right hemispheres).

Finally, for classes within which stimuli were familiar or not (classes II and III, see Fig. 1), no effect of familiarity could be observed: comparisons of unfamiliar, familiar and bird's own songs within classes II and III showed no significant difference between these stimuli, neither in the proportion of responding neuronal sites (see Fig. 3; two-way repeated-measures ANOVAs, p = 0.26 and 0.71 respectively for classes II and III) nor in the magnitude of the neuronal response (two-way repeated-measures ANOVAs, p = 0.21 and 0.60 respectively for classes II and III).

## Discussion

Our results show differential neuronal responses to ethologically defined classes of songs that have different functions and social values, in a non-primary, associative auditory area, both in the number of neuronal sites responding, and in the magnitude of the responses. Most importantly, these differential responses corresponded to the functional classes of songs, with increasing activation from non-specific to species-specific and from species-specific to individual-specific sounds. Our data thus provide a rare example of convergence between natural behaviour and neural activity, and point to a potential neural substrate for categorization of complex communication signals and vocal recognition of same-species members.

These results suggest that NCM could be the place for sorting sounds into categories in the songbird brain. Thus, although we did not (and could not) test a continuum of sounds from one category to another, the proportion of responding sites and the magnitude of responses clearly differed between functional classes of songs and less within them. Given the large structural variations within each class (e.g. between different individual-specific class-II whistles or warbling motifs; see Fig. 1), it is therefore very likely that NCM differential responses relied more on the stimuli behavioural salience than on their acoustic structure. Another element in this direction is the finding that clicks, which, contrarily to other warbling motifs, are common to all starlings, and which are normally produced within a sequence of other motifs, were processed similarly to the very dissimilar structures that are pure tones or white noise. Finally, the fact that familiarity did not modify within-class responses demonstrates a capacity of generalization, which is one of the cue properties of categorization.

Several authors have questioned the role of the NCM in the emergence of the highly complex and song-selective responses observed in higher-order song nuclei [16]–[22]. Here, we show that NCM neurons appear to differentially respond to distinct classes of songs corresponding to different levels of recognition (going from species-specific to individual-specific). Interestingly, the most effective stimuli at driving the neurons were the highly individual class-III motifs, which are thought to be the basic units of individual recognition in starlings [43], [44]. NCM neurons could therefore actually contribute to the processing resulting in the strong selectivity for both spectral and temporal properties of song observed in higher-order regions of the avian brain [45]–[47]. This selectivity is known to arise during development in neurons that are initially unselective [48]. One can therefore wonder what happens at the level of the NCM, and how the differential responses we observed develop. Evidence exists that NCM neuronal responses are shaped by the animal's prior experience with song [49]. Moreover, we have shown experience-dependent effects at the level of the main input to NCM (the Field L, which is comparable to the mammalian primary auditory cortex) [50], [51]. Given that, in a hierarchical scheme of sensory processing, plasticity at the primary levels should influence higher-order regions, it is very likely that NCM response properties are also expressly determined by the animal's unique experience. This will have to be confirmed by studying how these response properties develop, for example in birds lacking experience with one or several classes of songs for which we here observed differential neuronal responses. We already know that, in young starlings' vocalizations, some classes of songs can be absent according to the birds' experience [36], [38]. We also know that class-I songs are learned later in development [40]. Interestingly, this class of songs was the least effective natural class of songs in our study. We could therefore imagine that NCM responses may modulate input to the song system, thus playing the role of an “intrinsic perceptive filter” [52] and favouring learning of class-III songs first, class-II songs next, and finally class-I songs. Such a mechanism would be somewhat reminiscent of what is thought to happen in human infants, who may use the acquired phonological categories of the language to which they are exposed to guide speech development [53].

The strong similarities that the process of vocal imitation through which songbirds learn their vocalizations presents with the mechanisms underlying the ontogeny of human language have been known for long [9]–[11], and they have made songbirds the most relevant biological model to understand language acquisition and its neural correlates [54], [55]. Much more recently, a consortium of avian brain specialists has also unveiled similarities in the brain structures of birds and mammals [56], [57]. Some authors have thus compared NCM to the superficial layers of auditory cortex or to secondary mammalian auditory regions such as the lateral belt in primates or Wernicke's area in humans [14], [58]. We believe our study reinforces the parallel between these structures, and widen the impact of studies on songbirds. Indeed, we here observed differential representation of sounds with distinct biological significance (as observed in the field), as seen in higher-order fields of the auditory cortex of mammals, including humans. For example, differences in the biological significance of calls, as expressed in behavioural tests, can be seen in the activation of the secondary auditory field of mice [59]. Neurons in higher-order auditory cortical fields of monkeys prefer complex spectro-temporal acoustic patterns compared to simple sounds [60]. Finally, functional imaging studies in humans have shown a stronger activation in response to speech than in response to non-speech sounds in areas outside the primary auditory cortex in the temporal lobe [61]. Our results therefore show striking parallels with what has been observed in mammals, including humans, and point to an associative auditory area that provides starlings, and probably other higher vertebrates, with a potential neural substrate for the extraction of biologically relevant information contained in complex acoustic signals used in vocal communication. Overall, by indicating that NCM as a brain area is a likely candidate for sound categorization in birds, we believe that the present study improves our knowledge of the representation of sound significance in the songbird brain, and that it will participate in comparing sound categorization in vertebrates.

## Materials and Methods

### Experimental animals

Six wild-caught male starlings were used. Birds were caught as adults in Normandy (France) 2 years before the experiments, and were kept together in an indoor aviary with food and water ad libitum. Artificial light matching the natural photoperiod was provided.

After recording every bird's song repertoire in individual soundproof chambers, a stainless steel pin was attached stereotaxically to the skull with dental cement, under halothane anaesthesia. The pin was located precisely with reference to the bifurcation of the sagittal sinus. Birds were given a 2-day rest after implantation. From this time, they were kept in individual cages with food and water ad libitum. During the experiments, the pin was used for fixation of the head and as a reference electrode.

The experiment was performed in France (licence no. 005283, issued by the departmental direction of veterinary services of Ille-et-Vilaine) in accordance with the European Communities Council Directive of 24 November 1986 (86/609/EEC).

### Acoustic stimulation

In order to test responses of NCM neurons to both artificial non-specific sounds and natural species-specific sounds corresponding to distinct classes of songs, we used 23 stimuli including (Fig. 1):

- 6 artificial stimuli (0.5-, 1-, 2-, 4- and 8-kHz pure tones and white noise).
- 17 starling songs or song elements corresponding to the 3 classes described by Hausberger [28]:
  Class-I songs (universally shared male songs used in species-specific and population recognition) were represented by 2 exemplars of each theme described by Hausberger [28], recorded from a starling unknown to the experimental birds (unfamiliar dialectal variants) [see 29], [30].
  Class-II songs (used in individual recognition and social partnership) [31] included 2 exemplars from an unfamiliar bird, 2 exemplars from a familiar bird (a familiar bird was a bird that had been caught at the same time as the tested bird and that had spent 2 years in the same aviary), and 2 exemplars from the tested bird (bird's own songs).
  Class-III songs (also called warbling; used in individual recognition and in short-distance communication) included individual-specific motifs from an unfamiliar bird and from the tested bird, and one species-specific motif described in all studies on starling song (clicks) [32], [33].

Given that the class-I songs have been shown to be involved in vocal exchanges in a variety of contexts [29], and that, in the bird's repertoire, each class-II song seems to convey individual—and social—identity [31], [62], we chose to have a good representation of these songs in our stimuli. Therefore, the whole or most of the whistled-song (Class-I and -II songs) repertoire of each bird was represented in the stimuli. By contrast, given the high variability in starling Class-III songs and the limitations of stimulus repertoire sizes in electrophysiological experiments, we made no attempt to exhaustively represent the Class-III song material of each bird, and instead selected warbling motifs, which are thought to be the fundamental perceptual unit of individual vocal recognition in starlings [44].

The songs used were 398 to 1440 ms long (mean±SD = 927.5±314.4 ms), and the artificial stimuli were 100 ms long, with 20 ms rise and fall times. The 23 stimuli were randomly interleaved into a single sequence of stimuli that was repeated 10 times at each recording site. Note that, within this sequence, different exemplars of the same class usually followed exemplars from different classes, thus ensuring that the order in which the vocalizations were played to the birds (which was the same for every bird and every session) could not account for the observed pattern. The duration of the whole sequence of 23 stimuli was 30 s. The mean interval between stimuli was 802.5±399.2 ms (mean±SD), with a minimum of 300 ms. Although the inter-stimulus interval was reduced because of time limitations, stimulus presentation mimicked natural sequences of whistle production. Starlings tend to sing successions of whistles separated by 1 to 8 sec. Such sequences can include successions of up to 200 whistles, with repetitions of each whistle type in the repertoire. According to the social context, these successions of whistles may be followed by a sequence of continuous warbling or not [29], [63].

Auditory stimuli were delivered in an anechoic, soundproof chamber through a loudspeaker located 20 cm in front of the bird's head. The peak sound pressure at the bird's ears was 85 dB SPL for all the stimuli, which corresponded to a RMS of 65 dB.

### Data collection

The systematic approach used to record neuronal activity in the brain of our birds has been described in George et al. [64]. In brief, we used a linear array of 4 microelectrodes made of tungsten wires insulated by epoxylite, each spaced 625 µm apart (FHC n°MX41XBWHC1). Electrodes impedance was in the range of 3–6 MΩ.

**Figure 6:**
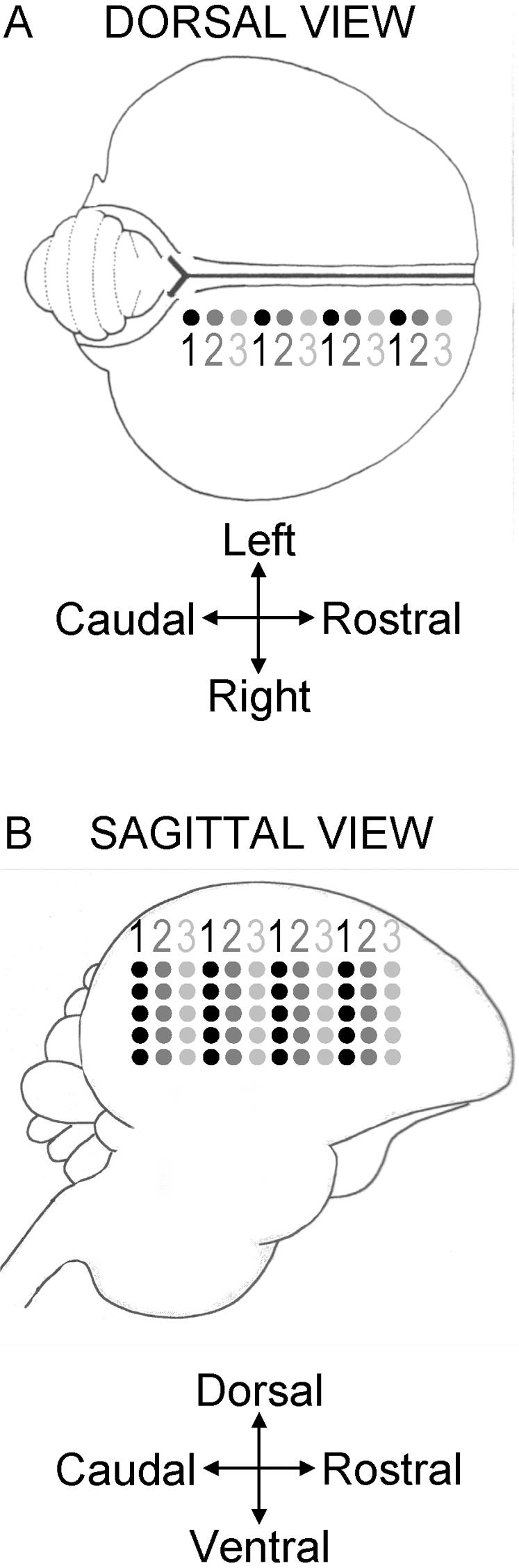
Schematic representation of the positions of the penetration and recording sites in one hemisphere (not to scale). The numbers below and above the dots indicate the recording sessions. A: Dorsal view. Black and grey dots indicate the penetration sites. B: Sagittal view. Black and grey dots indicate the recording sites. Modified from [64].

Recordings were performed in awake-restrained starlings, in one sagittal plane in each hemisphere, at 400–500 µm from the medial plane. Recordings in the left and right hemispheres were made alternately, at symmetrical locations. Each recording plane consisted of 1 to 3 penetrations systematically placed at regular intervals of about 230 µm in a rostrocaudal row (see Fig. 6), between 100–905 and 2496–3245 µm from the bifurcation of the sagittal sinus. In order to stay within the limits of the NCM, only the most caudal penetrations (that is less than 2000 µm from the bifurcation of the sagittal sinus) were kept for analyses. Despite this precaution, we cannot rule out the possibility that a minor fraction of our data derives from recordings outside of NCM. If so, however, we might have expected to observe differences in the pattern of response within the sagittal plane, but we failed to observe any such differences. Indeed, no particular pattern appeared with respect to the stimuli to which the sites responded (that is the same overall area was activated, whatever the stimulus or class of stimulus).

Given that we used a linear array of 4 microelectrodes, each plane was made of 4 to 12 electrode tracks. Only one session per day, lasting 3 to 4 h, was made in each bird. Data were thus collected over 2 to 6 days for each bird. Note that, between the recording sessions, birds were placed in individual cages, with food and water ad libitum, and a piece of plastic foam was placed over the skull opening in order to avoid any damage to the brain. Moreover, birds were weighed before each recording session, and their weight remained stable over the whole data collection.

Neuronal activity was recorded systematically every 200 µm, dorso-ventrally along the path of a penetration, independently of the presence or absence of responses to the stimuli we used, between 400 and 5600 µm from the surface of the brain. Among the recording sites, some clearly responded to the stimuli, others did not (see Results).

NCM neurons have been shown to habituate rapidly and selectively to species-specific songs, and this habituation appears to be long-lasting [16], [17]. However, we did not observe any such habituation effect in our recordings. This is very likely due to our protocol. Indeed, our recordings typically started 400–2000 µm below the surface of the brain, and responses began to be observed between 800–3800 µm (mean±SE = 1892.3±230.5 µm). This means that birds had already heard each stimulus at least 20 times before we began to record responses to our stimuli. According to Stripling et al. [19], electrophysiological responses to song change immediately and dramatically after the first stimulus presentation, and the spike rate declines slowly throughout the first 20–30 presentations of a stimulus but then stabilize at a level of about 60% of the initial response. It is therefore very likely that our recordings were performed during this stable phase of response.

All the data were collected between mid-February and mid-March, at a time when wild starlings are very actively singing. Note that, in starlings, the number of NCM cells expressing ZENK in response to song playback does not vary with sex or photoperiod [65].

### Histology

The anatomical locations of the recording sites were determined from reference marks consisting of Alcian blue injections made after the last recording session through a glass micropipette, at 4 defined locations within the recording plane of each hemisphere. Reference marks were made in the most anterior and posterior penetrations, at 2 different depths. The precise coordinates of these marks with reference to the bifurcation of the sagittal sinus and to the surface of the brain were known. At the end of the experiment, birds were given a lethal dose of urethane and perfused with 0.9% saline followed by 4% formaldehyde. Dye marks were located on 25 microns frozen sections stained with cresyl violet.

### Data analysis

At each recording site, before the stimulation, the experimenter manually controlled the amplitude discrimination in order to limit the recordings to the neuron exhibiting the biggest spikes, using a custom-made time- and level-window discriminator [see also 64]. Although it has been argued that the amplitude is the most effective feature for spike sorting [66]; see also 67,[68], we cannot rule out the possibility that a small fraction of our recording sites corresponded to multi-unit data consisting of a small cluster of 2–4 neurons. However, several studies found that analyses resulting from single and multi units were similar [69], [70].

The computer that delivered the stimuli also recorded the times of action potentials and displayed on-line rasters of the spike data for the 4 electrodes simultaneously. At each recording site, spontaneous activity was measured during 1.55 s before the presentation of the first stimulus of each sequence, which resulted in 10 samples of spontaneous activity (that is a total of 15.5 s).

The neuronal activity related to the 10 repetitions of the stimulus set, as well as the 10 corresponding samples of spontaneous activity, were subdivided into 50-ms time bins of activity, like in a previous study [71]. The activity level (number of action potentials) within every 50-ms period was calculated.

The spontaneous activity measured during the 1.55 s preceding the very first presentation of the stimulus sequence was used to determine the frequency of occurrence of action potentials at rest. For that, the 1.55-s sample was subdivided into 31 50-ms time bins, each containing a given number of action potentials. A binomial test was then used to determine whether the activity level in each bin during the whole duration of each stimulus plus up to 100 ms after it had ended (to account for latencies in the neuron's responses) significantly exceeded the spontaneous activity level observed during the 1.55 s preceding the first presentation of the stimulus sequence, at the 0.05 level of significance. Due to the low level of spontaneous activity, only excitatory responses were detected. Note that this first test was in terms of the probability of finding one or more action potentials in a bin [for more details, see 68]. For example, if the 1.55-s sample of activity preceding the very first presentation of stimulus sequence contained four 50-ms bins with 1 action potential, and 1 bin with 2 action potentials, the probability to obtain one spike in one bin was 0.13 (that is 4 divided by the total number of 50-ms bins = 31), and the probability to obtain two spikes in one bin was 0.03 (that is 1 divided by 31). In this case, all individual bins containing two or more spikes were therefore considered as significant activations. When a given number of spikes (e.g. two) showed a P≤0.05 while a higher number of spikes (e.g. three) had a P≥0.05, then the next higher number of spikes (e.g. four) exhibiting a P≤0.05 was considered as a reference to determine significant activation.

Then, each of the 10 stimulus repetitions was tested individually and the same bin (with respect to the stimulus) had to reach the responsiveness criterion independently over a significantly higher number of repetitions than expected by chance (binomial test at the 0.05 level of significance) to be considered as a reliable response to the stimulus. The number of repetitions reaching the responsiveness criterion expected by chance was determined from the 10 samples of spontaneous activity collected during the 1.55 s preceding each stimulus sequence and corresponded to the maximum number of activations observed in the same bin. For example, if the maximum number of trials without acoustic stimulation showing significant activation at the same time bin was one, it set P≤0.1. We then used the binomial distribution to find the number of trials k so that P(x≥k) ≤0.05, where x is the number of trials with acoustic stimulation showing significant activation at the same time bin. In the example given, k = 4. The range of the number of trials that were needed to find significance was four to eight. A recording site was classified as responsive if and only if the latter test was significant for at least one series of bin during a given stimulus.

Only responsive sites were further analyzed. For that, we calculated the proportion of sites responding to each stimulus, and to each class of stimuli (see Fig. 3 and 4). Moreover, to assay the strength of the neuronal responses, we used Z-scores (see Fig. 5). Z-scores are defined as the difference between the firing rate during the stimulus and that during the background activity divided by the standard deviation of this difference quantity [see 72]. This measure gives a good idea of the strength of the response, independently of the duration of the stimuli [see 67].

For statistical comparisons, we used the mean values calculated for individual birds. Two-way repeated-measures ANOVAs and PLSD Fisher tests (StatView 5.0 for Windows, SAS Institute Inc.) were performed to test for potential differences between the two hemispheres and the different classes of stimuli, independently for proportions of sites and Z scores. For the proportions of sites, data were normalized using an arcsin square-root transform. Unless otherwise indicated, data are presented as mean±standard error of the mean (SE).
